# Seeing Is Craving: Neural Dynamics of Appetitive Processing During Food-Cue Video Watching and Its Impact on Obesity

**DOI:** 10.3390/nu17152449

**Published:** 2025-07-27

**Authors:** Jinfeng Han, Kaixiang Zhuang, Debo Dong, Shaorui Wang, Feng Zhou, Yan Jiang, Hong Chen

**Affiliations:** 1 School of Psychology, Southwest University, Chongqing 400715, China; hanjinfengpsy@126.com (J.H.); debo.dong@gmail.com (D.D.); shaoruiwang@yeah.net (S.W.); fengzhou0@swu.edu.cn (F.Z.); jiangyan_12@126.com (Y.J.); 2 Institute of Science and Technology for Brain-Inspired Intelligence, Fudan University, Shanghai 200433, China; zhuangkx@fudan.edu.cn; 3 Key Laboratory of Cognition and Personality (Ministry of Education), Southwest University, Chongqing 400715, China; 4 Research Center of Psychology and Social Development, Southwest University, Chongqing 400715, China; 5 The Collaborative Innovation Team on Child and Adolescent Mental Health, Southwest University, Chongqing 400715, China

**Keywords:** food cues, appetite, digital media exposure, fMRI, eating behavior, obesity

## Abstract

**Background/Objectives**: Digital food-related videos significantly influence cravings, appetite, and weight outcomes; however, the dynamic neural mechanisms underlying appetite fluctuations during naturalistic viewing remain unclear. This study aimed to identify neural activity patterns associated with moment-to-moment appetite changes during naturalistic food-cue video viewing and to examine their relationships with cravings and weight-related outcomes. **Methods**: Functional magnetic resonance imaging (fMRI) data were collected from 58 healthy female participants as they viewed naturalistic food-cue videos. Participants concurrently provided continuous ratings of their appetite levels throughout video viewing. Hidden Markov Modeling (HMM), combined with machine learning regression techniques, was employed to identify distinct neural states reflecting dynamic appetite fluctuations. Findings were independently validated using a shorter-duration food-cue video viewing task. **Results**: Distinct neural states characterized by heightened activation in default mode and frontoparietal networks consistently corresponded with increases in appetite ratings. Importantly, the higher expression of these appetite-related neural states correlated positively with participants’ Body Mass Index (BMI) and post-viewing food cravings. Furthermore, these neural states mediated the relationship between BMI and food craving levels. Longitudinal analyses revealed that the expression levels of appetite-related neural states predicted participants’ BMI trajectories over a subsequent six-month period. Participants experiencing BMI increases exhibited a significantly greater expression of these neural states compared to those whose BMI remained stable. **Conclusions**: Our findings elucidate how digital food cues dynamically modulate neural processes associated with appetite. These neural markers may serve as early indicators of obesity risk, offering valuable insights into the psychological and neurobiological mechanisms linking everyday media exposure to food cravings and weight management.

## 1. Introduction

Food-cue processing involves the cognitive, emotional, and neural responses elicited when individuals encounter food-related stimuli through visual, olfactory, or auditory channels [1]. This fundamental neurocognitive process critically modulates eating behavior and body-weight regulation by influencing reward valuation, attentional biases, and decision-making underlying food selection [2]. The rapid proliferation of digital media has substantially increased daily exposure to food-related visual content, potentially affecting dietary decisions both immediately and over time [3,4]. Given these evolving circumstances, investigating the neural mechanisms that underlie food-cue processing within authentic, real-world environments has become essential for advancing our understanding of modern eating behaviors.

Prior neuroimaging research consistently highlights significant individual differences in food-cue processing, particularly associated with weight status. For instance, individuals with overweight or obesity typically exhibit greater neural activation within reward-related regions, such as the striatum and orbitofrontal cortex, in response to visual food stimuli compared to healthy-weight counterparts [5,6]. This heightened neural responsivity reflects increased sensitivity to food cues, potentially promoting higher appetite, stronger cravings for energy-dense foods, maladaptive eating patterns, and excessive caloric intake [7,8]. Meta-analytic evidence further demonstrates a positive relationship between food-cue reactivity and Body Mass Index (BMI), identifying elevated reactivity as a robust predictor of weight gain and obesity risk [1,9]. Such associations are increasingly relevant in contemporary media environments, where individuals face unprecedented and persistent exposure to food stimuli via digital platforms and targeted advertising [10]. Collectively, these findings underscore the critical role of individual variability in food-cue responsivity as a neurocognitive mechanism underlying overeating and obesity.

However, existing research predominantly employs categorical comparisons (e.g., high-versus low-calorie stimuli) [11,12], leaving critical questions unanswered regarding how neural responses continuously fluctuate with appetite and how such temporal dynamics influence subsequent eating behaviors and weight-related outcomes. Additionally, most experimental paradigms rely on static food images, limiting ecological validity and failing to capture the dynamic, multimodal, context-rich nature of food cues encountered in daily life [13]. Importantly, food-cue processing itself unfolds dynamically, involving moment-to-moment shifts in attention, reward valuation, and inhibitory control across multiple cognitive stages [14]. To better approximate real-world eating behaviors, adopting dynamic, continuous analytical approaches is thus essential.

Recent methodological advances have emphasized the utility of naturalistic stimuli, particularly video-based paradigms, to enhance ecological validity in food-cue research [15]. Videos effectively capture the dynamic, context-rich properties of food encounters, closely mirroring contemporary digital consumption patterns [3,4,10]. Relative to static images, food videos evoke richer neural responses and complex network interactions, offering enhanced flexibility for manipulating stimulus attributes and enabling a precise examination of appetite fluctuations across specific temporal intervals [16,17].

Capturing these complex neural dynamics necessitates analytical strategies beyond traditional general linear model (GLM) approaches, which often struggle to model time-varying cognitive states. Hidden Markov Modeling (HMM), a sophisticated time-series approach, offers distinct advantages by identifying latent neural states that characterize whole-brain activation patterns and interregional connectivity across time [18,19]. Recent evidence demonstrates that HMM-derived brain states possess superior reliability and predictive validity compared to traditional methods [20]. Furthermore, HMM has successfully captured neural dynamics underlying complex cognitive processes such as memory retrieval and decision-making [21].

Within appetitive contexts, HMM-derived states may effectively characterize sustained cognitive experiences (e.g., imagined consumption), providing insights into individual differences in food-cue processing based on BMI [22]. Indeed, recent large-scale neuroimaging studies have revealed robust associations between BMI and resting-state neural dynamics [23]. Extending these findings, we hypothesize that food-cue-evoked neural state transitions exhibit distinct BMI-dependent patterns, offering mechanistic insights into differential food-cue processing across weight categories.

In the present study, we combined ecologically valid digital stimuli (online food-related videos) with continuous appetite ratings to characterize dynamic neural states underlying food-cue processing using HMM and machine learning regression analyses. This approach addresses a critical gap in existing literature, which has predominantly relied on static stimuli and categorical analytical frameworks, limiting our understanding of the continuous, dynamic nature of appetite regulation in real-world digital environments. Specifically, we investigated how momentary appetite fluctuations modulate neural dynamics during food-cue exposure, examined the influence of weight status (healthy weight vs. overweight/obese) on these neural patterns, and assessed their associations with immediate subjective food cravings. Additionally, we explored whether these appetite-related neural dynamics predict longitudinal BMI trajectories over six months. Our approach provides novel mechanistic insights into how digital food environments dynamically shape neural processing, consumer preferences, and obesity risk over time.

## 2. Materials and Methods

### 2.1. Participant

Two independent groups of participants were recruited for the experiment; one group (appetite rating group; *N* = 40 females; mean age ± standard deviation: 20.50 ± 1.79 years) actively rated their appetite in response to the video materials containing food cues, while the other group (fMRI scanning group; *N* = 58 females; 20.76 ± 1.53 years) passively watched the same videos during an fMRI scan and completed a subsequent series of experiments. Due to ineffective key responses, such as incorrect key presses, by two raters, data from 38 raters were retained for the final analysis. However, data from all 58 participants who underwent fMRI scanning were included in the analysis. To minimize the potential influence of gender differences on appetitive processing, this study included only female participants. Prior studies indicated that females are more reactive to visual food stimuli compared to males [24,25].

All participants were right-handed, had normal or corrected-to-normal vision, and were screened to exclude color blindness or color deficiency. None of the participants reported a history of physical illnesses or mental disorders, as defined by the Diagnostic and Statistical Manual of Mental Disorders, Fifth Edition (DSM-5) [26]. Additionally, participants were evaluated for eating disorders using the Eating Disorder Diagnostic Scale (EDDS), a well-established 22-item screening tool based on the DSM criteria for eating disorders [27]. The EDDS assesses key symptoms associated with anorexia nervosa (AN), bulimia nervosa (BN), and binge eating disorder (BED). Participants scoring above the clinical threshold of 16.5 on the EDDS or self-reporting a history of food addiction were excluded from this study. Prior to participation, written informed consent was obtained from all individuals. The study protocol was approved by the institution’s local ethics committee and adhered to the principles outlined in the Declaration of Helsinki. Participants received monetary compensation for their time and effort.

### 2.2. Critical Video Stimulus and Appetite Ratings

**Figure 1:**
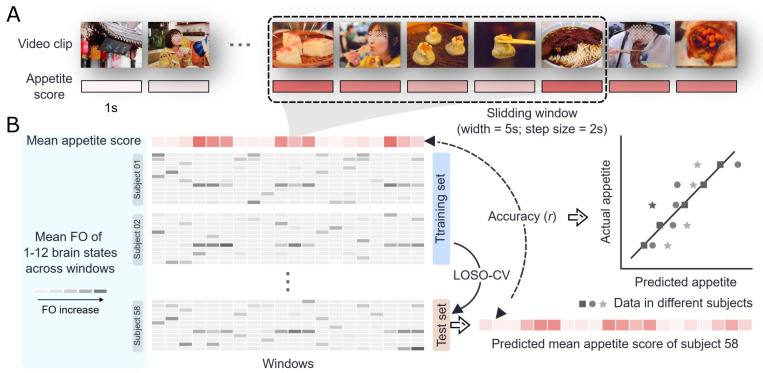
Illustration of the prediction framework for appetite scores during video viewing. (**A**) The appetite scores within each video are sampled at 1-s intervals and averaged using a sliding time window with a width of 5 s and a step size of 2 s. (**B**) Ridge regression is applied to predict appetite scores based on brain state fractional occupancy (FO) values, with a cross-validation framework implemented to evaluate prediction performance.

The experimental paradigm employed two continuous video stimuli containing food-related cues (Figure 1A; duration: 6 min 29 s and 4 min 44 s, respectively). These stimuli comprised systematically alternating sequences of appetite-eliciting content (featuring eating behaviors and visually appealing food presentations) and control content (natural landscapes and street views devoid of food cues), presented in a naturalistic, continuous manner. The presentation sequence of videos was counterbalanced across participants to mitigate potential order effects. Stimulus materials were selected from reputable video-sharing platforms (Bilibili and YouTube), focusing on relevant content categories such as food, lifestyle, and documentaries to ensure ecological validity and alignment with the study objectives. All acquired content was then processed using CapCut video editing software (V3.2.0) to remove advertisements and ensure a balanced ratio of food and non-food stimuli. To control for potential confounding influences of auditory processing, all audio components were systematically removed. The final stimuli were standardized in MP4 format with high-definition specifications (resolution: 1920 × 1080 pixels; frame rate: 60 Hz).

To assess the appetite level elicited by food cues in the videos, participants in the appetite rating group were instructed to provide real-time ratings during video viewing. The experiment was conducted in a controlled behavioral psychology laboratory equipped with desktop computers of identical specifications. The brightness, contrast, color settings, and other display parameters of all computers were standardized by the experimenter. Video playback and presentation were centrally controlled using a program developed with MATLAB’s Psychtoolbox-3 (PTB-3, version 3.0.19.0). Each participant viewed the video independently on a single computer, sitting 0.5 m away from the screen, with the video displayed in full-screen mode. Prior to the experiment, participants received standardized instructions from the experimenter to ensure a clear understanding of the procedure. During video exposure, participants continuously rated their momentary appetitive states on a three-point ordinal scale (1 = mild appetite, 2 = moderate appetite, 3 = strong appetite). If no desire to eat was elicited by the video content, participants did not press any key. Ratings were made using the numeric keypad on the right side of the keyboard, with participants instructed to use their right hand for responses.

To evaluate the reliability of appetite ratings, we conducted a split-half consistency analysis [28]. First, the data were randomly divided into two non-overlapping halves, and the mean appetite ratings for food cues in the two videos were calculated separately for each group of participants. Next, we computed the rank-order Spearman correlation of appetite scores across time points between the two halves and applied a Spearman-Brown correction to derive the split-half correlation coefficient. This procedure was repeated 5000 times, and the mean correlation coefficient across iterations was used as the representative measure of split-half reliability. To assess statistical significance, we generated a surrogate null distribution by calculating an additional rank-order correlation coefficient for each iteration after randomly shuffling the appetitive rating ranks from one of the split halves. The observed split-half reliability measure (mean correlation coefficient) was subsequently compared to the null distribution to calculate a right-tailed *p*-value. This *p*-value reflects the probability of obtaining an average split-half correlation coefficient from the observed data that exceed the values in the null distribution, assuming the null hypothesis is true (i.e., zero correlation in the shuffled data).

### 2.3. Validation Video Materials

A core objective of this study was to predict appetite levels elicited by the videos based on dynamic brain features. However, high-appetite scenes often draw greater attention (e.g., participants may focus more on food-related images than on landscapes) and may evoke stronger emotional valence and arousal (e.g., content creators displaying more joyful expressions during appetite-related scenes). To ensure that the identified brain features were attributable to appetite levels rather than other confounding factors, we collected an additional set of video stimuli using a blocked design to validate the core findings.

We selected three categories of short videos as validation stimuli: (1) Mukbang, featuring individuals actively consuming food to provide strong appetite-related cues; (2) Food presentation videos, showcasing food items without individuals or eating behaviors to isolate the visual appeal of food; and (3) Sport videos, depicting physical activities to control for high arousal and attention without eliciting appetite responses. The source and editing specifications of the video materials were consistent with those of the two longer videos. Five clips per category were included, resulting in a total of 15 short video clips (mean duration ± standard deviation: 59.38 ± 8.80 s). The experiment consisted of five runs, each containing three short videos from different categories, with the presentation order randomized across participants. After completing the fMRI scan, participants were asked to rate each video they had watched on three dimensions: “desire to eat,” “emotional valence,” and “emotional arousal.” Ratings for “desire to eat” ranged from 1 (very low desire to eat) to 5 (very high desire to eat), “emotional valence” ranged from 1 (very negative) to 5 (very positive), and “emotional arousal” ranged from 1 (very calm) to 5 (very excited).

### 2.4. Body Mass Index (BMI) Measurement and Categorization

To explore the potential relationship between neural dynamics during food-cue processing and BMI, BMI was assessed for all participants in the fMRI scanning group. Body Mass Index (BMI) was calculated as weight (kg) divided by height squared (m^2^). Following the classification standards of the World Health Organization (WHO), the 58 participants were categorized into two groups, an overweight/obese group (*N* = 29; BMI ≥ 25; mean BMI ± standard deviation: 26.89 ± 1.60, age: 20.34 ± 1.23 years) and a healthy-weight group (*N* = 29; BMI < 25; 20.39 ± 1.21; age: 21.17 ± 1.69 years). Participants’ height and weight were measured prior to the MRI scan. Weight and body composition were measured using a multi-frequency bioelectrical impedance analyzer (Seca^®^ Medical Body Composition Analyzer 515/514; seca GmbH & Co. KG, Hamburg, Germany). The Seca 515/514 is an 8-point analyzer equipped with contact electrodes integrated into a standing platform and attached handrails. Participants stood barefoot on the analyzer’s platform and grasped the handrail electrodes during measurement. Body weight was automatically measured by the analyzer’s integrated electronic scale with an accuracy of 0.1 kg. Height was measured separately to the nearest 0.1 cm using a calibrated stadiometer.

In addition to the baseline BMI measurement (conducted on the same day as the MRI scan), each participant’s BMI was tracked over a six-month period, with measurements taken monthly from April to September. Due to one participant not taking part in the follow-up, baseline BMI measurements were obtained for all 58 participants, while longitudinal BMI data across six time points were collected for 57 participants. To categorize the BMI change trajectories, we conducted a clustering analysis on the BMI change matrix of 57 participants. The clustering process involved three key steps. First, we constructed a correlation matrix to quantify the similarity in inter-individual BMI change patterns over six data points. Next, hierarchical clustering with the average linkage method was applied to generate a dendrogram structure. Finally, cluster assignments were determined by cutting the dendrogram at the level corresponding to two clusters. This method effectively partitioned participants into two distinct groups with similar BMI change trajectories. The decision to select two clusters was supported by the Davies–Bouldin index, which exhibited a local minimum, and the Calinski–Harabasz index, which reached a peak value, both indicating optimal cluster separation and cohesion at this level.

### 2.5. fMRI Experiment Procedure

The experimental protocol comprised two fMRI sessions. Participants were required to maintain a minimum 3-h fasting state prior to the initial session. Upon arrival at the imaging facility, standardized self-report measures of subjective hunger (“What is your current hunger level?”) and food craving (“How strong is your current desire to eat?”) were administered using an 8-point Likert-type scale [29]. Anthropometric measurements (height and weight) were subsequently obtained. Preliminary analyses of pre-scanning data revealed non-significant correlations between BMI and food craving (*r* = 0.063, *p* = 0.645) or hunger ratings *(r* = −0.235, *p* = 0.084). These null associations provide evidence that the post-exposure variations in food craving observed in this study can be attributed to the experimental manipulation of food cue exposure rather than to pre-existing individual differences in anthropometric characteristics or baseline motivational states.

During the first scanning session, participants lay in a supine position with their heads immobilized and viewed video stimuli presented on a screen via a mirror attached to the head coil. The videos were displayed using an MRI-compatible projection system. The scanning protocol included the following sequences: (1) Resting-state functional scan: Participants were instructed to rest with their eyes open, remain awake, and avoid engaging in specific thoughts. (2) Structural scan: Participants rested with their eyes closed while keeping their heads completely still. (3) Naturalistic video-viewing scans: Participants passively viewed two consecutive food-cue video stimuli without providing any responses. Between the two videos, participants were given the option to take a short break (no longer than 5 min) or proceed directly to the next video. After the scan, participants’ current levels of food cravings and hunger were also assessed. Analysis revealed a significant positive association between BMI and post-viewing food craving scores (*r* = 0.29, *p* < 0.05), while post-viewing hunger ratings showed no significant relationship with BMI (*r* = −0.03, *p* = 0.836).

The second scanning session was conducted one month after the first session. The pre-scan procedure was identical to that of the first session. During the scan, participants passively viewed three types of short videos. After the scan, participants rated the videos on three dimensions: “desire to eat,” “emotional valence,” and “emotional arousal.”

### 2.6. fMRI Data Acquisition and Preprocessing

All neuroimaging data were acquired using a 3T Siemens Prisma MRI scanner (Siemens Medical Systems, Erlangen, Germany) at the Brain Imaging Center of Southwest University. Functional MRI data during the task were collected using a multiband accelerated T2 *-weighted gradient-echo echo-planar imaging (EPI) sequence with the following parameters: repetition time (TR) = 1000 ms, echo time (TE) = 30 ms, flip angle (FA) = 73°, field of view (FOV) = 195 × 195 mm^2^, number of slices = 56, slice thickness = 2.5 mm, and isotropic voxel size = 2.5 × 2.5 × 2.5 mm^3^. High-resolution anatomical images were obtained with a three-dimensional T1-weighted magnetization-prepared rapid gradient-echo (MPRAGE) sequence: TR = 2530 ms, TE = 2.98 ms, FA = 7°, FOV = 256 × 256 mm^2^, number of slices = 192, slice thickness = 1.0 mm, and voxel dimensions = 0.5 × 0.5 × 1.0 mm^3^.

The MRI data were converted to the Brain Imaging Data Structure (BIDS) format [30] using the BIDScoin software (V4.5.0) [31]. Functional MRI (fMRI) data underwent extensive preprocessing using the fMRIPrep (version 23.2.0) toolchain [32], which is encapsulated within a Docker environment. Structural T1-weighted images underwent intensity normalization, skull-stripping, and brain tissue segmentation using fMRIPrep’s integrated preprocessing pipeline, which employs a hybrid skull-stripping approach combining ANTs-based methods and FreeSurfer algorithms for an accurate removal of non-brain tissues. Functional data were corrected for head motion artifacts using tools integrated within fMRIPrep, estimating six motion parameters (three translational and three rotational vectors). Motion-corrected functional images were resampled directly to the standard Montreal Neurological Institute (MNI) space for subsequent statistical analyses. Precise coregistration between functional images and corresponding T1-weighted structural images was performed using FreeSurfer’s boundary-based registration tool (bbregister). Spatial normalization to the standard Montreal Neurological Institute (MNI) space was achieved by applying nonlinear transformations computed from structural images using ANTs’ SyN algorithm, a state-of-the-art method for precise spatial alignment. The alignment leveraged the ICBM152 Nonlinear Asymmetric template (version 2009c).

Following basic preprocessing, noise in the unsmoothed functional imaging data was removed using the aCompCor algorithm [33], a widely used denoising approach designed to reduce physiological and scanner-related confounds. This method involves regressing out principal components derived from signal fluctuations within white matter and cerebrospinal fluid (CSF) masks, which are anatomically defined during preprocessing. Additionally, motion-related regressors, including the six rigid-body motion parameters (three translational and three rotational), their temporal derivatives, and their squared terms, were included in the nuisance regression model to account for motion-induced artifacts. Linear trends in the time series were removed to correct for scanner drift, and temporal band-pass filtering (0.008–0.09 Hz) was applied to retain neural signals within the frequency range of interest, while suppressing low-frequency drift and high-frequency noise.

### 2.7. Brain State Decomposition

After preprocessing, time-series signals were extracted using the 100-parcel cortical template from the Schaefer-2018 atlas [34], a high-resolution functional connectivity-based parcellation widely used in fMRI studies. For each parcel, the mean signal across all voxels was calculated to generate its time series during the viewing of the two long videos. The time series for each participant was then standardized to zero mean and unit variance, removing inter-individual differences in signal amplitude. This standardization allowed comparison across participants and captured the fluctuation patterns of each parcel relative to its own mean during the long video session.

The Hidden Markov Model (HMM) was applied to fMRI time series to decompose the underlying brain states during video viewing. The HMM allows continuous BOLD signal fluctuations to be segmented into a series of discrete latent brain states, which dynamically switch and recur over time according to specific transition probabilities. Consequently, at each time point during video viewing, brain activity can be classified into a specific state, reflecting the average cortical activity pattern associated with that state [35]. To enhance the robustness of state estimation and ensure the consistency of state definitions across participants, the standardized time series from all participants were concatenated along the temporal axis to form a single, continuous time series as input for HMM modeling [20,36].

To reduce the number of parameters in state estimation, the time series underwent preprocessing before HMM training [37]. Principal component analysis (PCA) was applied to the demeaned, standardized, and concatenated time series, resulting in 49 principal components that captured approximately 90% of the signal variance. This step improved the signal-to-noise ratio, enabling more reliable state inference. The HMM parameter estimation was conducted using a variational Bayes framework, which minimizes free energy to optimize the model [38]. Modeling and inference were performed using the HMM-MAR toolbox developed by the Oxford Centre for Human Brain Activity (OHBA) (https://github.com/OHBA-analysis/HMM-MAR, accessed on 15 January 2024).

It is important to note that the total number of brain states (K) was a free parameter that needed to be determined prior to HMM inference. To identify the optimal number of brain states, we followed previous studies [19,20,36] and evaluated models with K ranging from 5 to 12 states based on their stability [23]. Specifically, for each K value, participants were randomly divided into two subsets, and HMM inference was performed independently on each subset. The model trained on one subset was then generalized to the other subset by assigning brain states to each time point (TR) in the test set based on the trained model from the training set. This procedure was repeated 10 times. If a model with a given K value exhibited strong generalizability and was robust to random sampling, the state assignments of models trained independently on the two subsets should show high consistency when applied to the same dataset. To evaluate this consistency, we used three metrics from different perspectives to assess model similarity: (1) zRand index: zRand is a standardized version of the Rand index, specifically designed to compare the similarity between two sets of clustering results. (2) Activity concordance index (CI): The CI measures the similarity of activation patterns for the same brain state across the two independent datasets. (3) Normalized mutual information (NMI): Based on information theory, NMI quantifies the shared information between two clustering solutions. These metrics provided complementary insights into the reliability and robustness of the brain state models across different K values.

### 2.8. Alignment of Appetite Ratings with Brain States

To link appetite ratings with brain states, the continuous appetite scores from all participants in the appetite rating group were resampled to match the TR (1 s). Specifically, scores were averaged within each TR and then z-score standardized along the temporal dimension. The group-level time series, representing fluctuations in appetite over time, was obtained by averaging the standardized appetite ratings across all participants at each time point. To account for the inherent time delay between neural activity and blood-oxygen-level-dependent (BOLD) signal changes, the group-level appetite rating time series was convolved with a hemodynamic response function (HRF). This step corrected for the temporal lag between neural activity and its BOLD representation, allowing subsequent analyses to more accurately capture the relationship between brain activity and appetite ratings [39]. We then applied a sliding window approach to align behavioral data with brain state dynamics on a larger temporal scale. Specifically, the window length was set to 5 TRs with a sliding step of 2 TRs, resulting in 194 and 142 time windows for the two videos, respectively. For each time window, the average appetite rating and the fractional occupancy (FO) of each brain state were calculated. FO represents the proportion of time a specific brain state is active within a given observation period [19].

### 2.9. Prediction of Appetite Scores Based on Brain States

We employed ridge regression to construct a predictive model for appetite scores based on brain state FO values (Figure 1B). Ridge regression addresses multicollinearity by introducing an L2 penalty term (λ) to the loss function, which shrinks model coefficients to prevent overfitting and stabilize predictions [40]. The accuracy and generalizability of the model were validated using a cross-validation framework.

**Model accuracy assessment.** The accuracy of the model was assessed through a nested “Leave-One-Subject-Out” cross-validation (LOSO-CV) approach. In the outer cross-validation (outer-CV) loop, the ridge regression model was trained on data from 57 participants and tested on the remaining participants’ data, which included either 194 or 142 data points (depending on the video). This process was repeated 58 times to ensure that every participant served as the test subject once. Within each fold of the cross-validation process, we first regressed out age, sex, and head motion effects from each feature (FO values) in the training dataset using a simple linear model. Then, the training data were normalized to the 0–1 range, and the same scaling parameters were applied to the test data. This accuracy evaluation was conducted separately for data from two independent videos.

**Model parameter optimization.** Within each iteration of the outer-CV loop, an additional five-fold inner cross-validation (inner-CV) loop was applied to optimize the ridge regression’s hyperparameter λ. Twenty candidate λ values (logarithmically spaced between 0.001 and 1000) were tested. The optimal λ was selected based on a joint optimization criterion that considered both prediction accuracy and model stability [41]. Accuracy was quantified as the mean Pearson correlation between the true and predicted values across the five inner-CV repetitions. Stability was assessed as the average Pearson correlation of model weights across the five folds, further averaged over the five inner-CV repetitions. Both accuracy and stability were scaled to the 0–1 range, and a joint metric was calculated as the Euclidean distance from the ideal point (1, 1). The λ value with the smallest distance was selected as the optimal parameter in each inner-CV. After determining the optimal λ, the model was retrained on the full training dataset using this parameter and applied to predict the test set’s target variable (appetite scores). The group-level optimal λ was determined by averaging the parameter performance, measured as the Euclidean distance, across all iterations.

**Model generalizability assessment.** We evaluated the model’s generalizability by testing its predictive performance on entirely independent video datasets. Specifically, the model was trained on all data from one video (using the group-level optimal λ) and then applied to predict appetite scores in the other video’s dataset. This cross-dataset generalization process was conducted in both directions, alternating between the two videos.

**Feature importance assessment.** To identify FO features that consistently contributed to the predictive model, we conducted a bootstrap test of feature importance [42]. The prediction procedure was repeated on 5000 bootstrap samples using the averaged group-level optimal λ. For each feature, the mean and standard deviation of the ridge regression coefficients across the bootstrap samples were used to calculate Z-scores and two-tailed *p*-values. To control for multiple comparisons, we applied a Benjamini-Hochberg false discovery rate (FDR) correction (*q* < 0.05) across all features. The absolute value of the Z-score reflected each feature’s stability in contributing to the predictive model, while the sign of the Z-score indicated the direction of the relationship between the feature and the target variable.

**Feature interpretation.** We characterized the neurobiological features of brain states that significantly contributed to prediction, focusing on cortical activation and functional connectivity patterns. To investigate the cognitive associations of the most highly activated regions, we employed the NiMARE (Neuroimaging Meta-Analysis Research Environment) toolbox for reverse inference decoding [43]. Specifically, the ROI-based decoder was applied to the top 10% most highly activated regions of interest (ROIs) to identify cognitive terms and functions statistically linked to the observed activation patterns, using the large-scale meta-analytic NeuroSynth database [44]. The decoding process utilized a Chi-square test to evaluate the co-occurrence of activation in the input ROIs with specific cognitive terms across the database. This analysis generated a ranked list of cognitive terms, each accompanied by a Chi-square statistic and corresponding *p*-value. To ensure reliability, we applied FDR correction for multiple comparisons. For interpretability, the decoded terms were further restricted to those present in both the NeuroSynth database and the Cognitive Atlas [45], ensuring alignment with established cognitive ontologies [46].

### 2.10. Statistical Analysis

The strength of the association between actual and predicted appetite scores, as well as the correlations among other continuous variables, was assessed using Pearson correlation. Independent sample *t*-tests were conducted to compare group differences. One-way repeated measures ANOVA was employed to evaluate differences in neural characteristics across conditions in short movies. Multiple comparisons were corrected using the false discovery rate (FDR). To evaluate whether state expression functioned as a mediating variable in the relationship between BMI (*X*) and food craving (*Y*), we implemented multilevel two-path mediation analyses using the Mediation Toolbox [47]. For statistical inference, we employed bias-corrected, accelerated bootstrap tests with 10,000 iterations, which is recommended for mediation analyses to address non-normally distributed sampling distributions of indirect effects. Mediation was determined to be statistically significant at *α* = 0.05 if the 95% confidence interval of the bootstrapped indirect effect (*a* × *b*) excluded zero.

## 3. Results

### 3.1. Food-Cue Videos Elicited Temporally Consistent Appetite Ratings Across Participants

To investigate the neural dynamic features associated with appetite level during naturalistic food-cue processing, we recruited two groups of female participants who viewed two continuous videos containing food cues. Each video contained numerous stimuli that were likely to elicit appetite responses in participants (e.g., eating behaviors and visually appealing food segments), interspersed with scenes of natural landscapes and street views that were unrelated to food cues. These two types of stimuli were presented in a natural and continuous manner, alternating with each other throughout the videos. The presentation order of the two videos was randomized for each participant. Split-half analyses revealed that the group-level appetite scores were consistent across both videos (video 1: *r* = 0.797, bootstrapped 95% confidence interval [CI] = [0.795, 0.798], *p* < 0.0001, one-tailed; video 2: *r* = 0.704, 95% CI = [0.702, 0.706], *p* < 0.0001; Supplementary Figure S1). This finding indicates that the appetite levels elicited by different segments of the videos are highly consistent across participants.

### 3.2. Dynamic Brain Features Reliably Predict Appetite Levels

To identify neural dynamics associated with appetite levels, we recruited an independent scanning group (*N* = 58) who passively viewed identical videos during fMRI without performing ratings. This separation of rating and scanning procedures prevented potential confounds from neural activity unrelated to food-cue processing that might otherwise be induced by motor responses (e.g., button presses) during scanning. The HMM analysis was applied to BOLD time series extracted from the Schaefer-2018 parcellation atlas [34] to identify discrete, latent brain states during naturalistic video viewing. This approach segmented continuous neural signals into distinct latent states that transition probabilistically over time [35], allowing each timepoint to be classified into a specific neural state with characteristic cortical activation patterns. Following established protocols [23], we evaluated models with K = 5–12 states based on stability metrics, ultimately selecting K = 12 for optimal decomposition (Supplementary Figure S2). These states represent recurrent, structured patterns of functional activation across both video stimuli (Supplementary Figure S3).

We then employed a sliding window approach to align behavioral data with brain state dynamics at a broader temporal scale. Windows of 5 TRs with a sliding step of 2 TRs generated 194 and 142 time windows for the two videos. For each window, we calculated the mean appetite rating and the fractional occupancy (FO) of each brain state, which represents the proportion of time a state remains active within the observation period [19]. This approach addresses the inherent temporal lag in appetite processing, which integrates sensory, memory, and reward systems rather than responding instantaneously to stimuli [48,49]. When food stimuli appear, related information is retrieved from memory, associated with reward value, and often persists beyond stimulus presentation through sustained activation of reward pathways [50,51]. The sliding window technique provides temporal smoothing that effectively captures these delayed processing dynamics.

**Figure 2:**
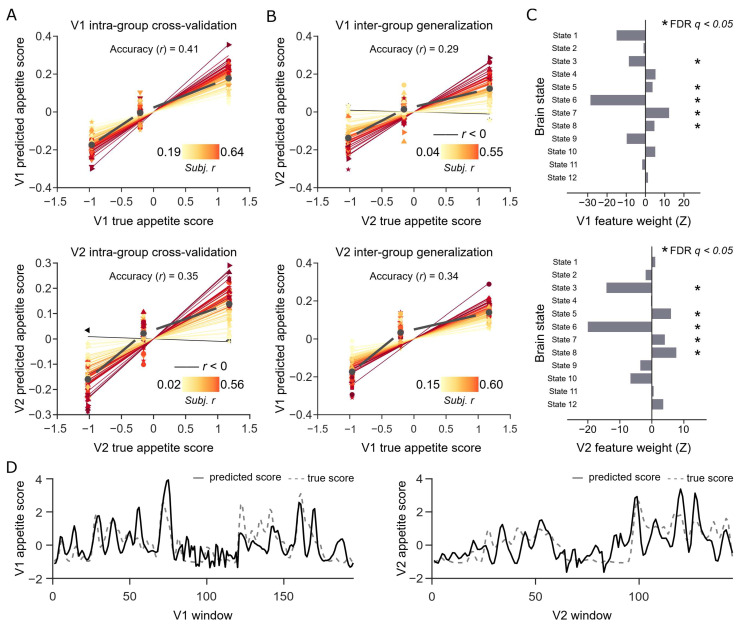
Performance of the prediction model for appetite scores. (**A**) Model accuracy assessment based on cross-validation within the two video datasets. The *x*-axis and *y*-axis represent the true and predicted appetite scores in Video 1 (V1) and Video 2 (V2), respectively. The values are centered by subtracting the mean of each participant. The data for each participant are divided into three bins, and the average true and predicted values within each bin are calculated and plotted. The black dots represent the averages. Each line represents the linear trend between the true and predicted values for each participant, with the strength of the correlation depicted by a color gradient. (**B**) Inter-dataset validation for assessing model generalizability on two video datasets. (**C**) The weight of each state feature in the prediction model. The weights are defined by the Z-statistics derived from the bootstrap distribution (FDR, *q* < 0.05). States that are significant in both datasets and have consistent weight directions are marked with an asterisk. (**D**) True and predicted appetite scores over the time series. The predicted appetite scores for each time window are generated based on the selected state features (those significant in both videos). The FO values of all participants’ states are averaged within each window. The predicted scores are defined as the dot product of the average FO values and the model weights.

We employed ridge regression to construct a predictive model for appetite scores based on brain state FO values. The accuracy and generalizability of the model were validated using a cross-validation framework. The cross-validation results within the videos indicate that the proportion of brain states (i.e., FO) within the time windows successfully predicted the appetite level of the window’s content; for Video 1, individual-level *r* = 0.41 ± 0.01 (mean ± standard error), group-level *r* = 0.41; for Video 2, individual-level *r* = 0.35 ± 0.02, group-level *r* = 0.35 (Figure 2A). The prediction model based on brain states was not constrained to specific video materials; models trained on one video can effectively generalize to another. That is, the model trained on Video 1 successfully predicted data from Video 2: individual-level *r* = 0.29 ± 0.02, group-level *r* = 0.29. Similarly, a model trained on Video 2 predicted data from Video 1: individual-level *r* = 0.34 ± 0.01, group-level *r* = 0.20 (Figure 2B). These findings suggest that individuals’ processing of appetite-related information in response to food cues is reflected in the dynamic patterns of their brain states.

We further analyzed the contribution of different brain states to the prediction model using a bootstrap method (Figure 2C). Notably, we identified five brain states that were significant in both video models (FDR, *q* < 0.05). These selected state features well predicted the appetite scores of the video content at the group-averaged level (Figure 2D; video 1: *r* = 0.67, video 2: *r* = 0.66). To further confirm that the results were not influenced by the sliding window length or step size, we also validated the findings using alternative window parameters (Supplementary Figure S4).

**Figure 3:**
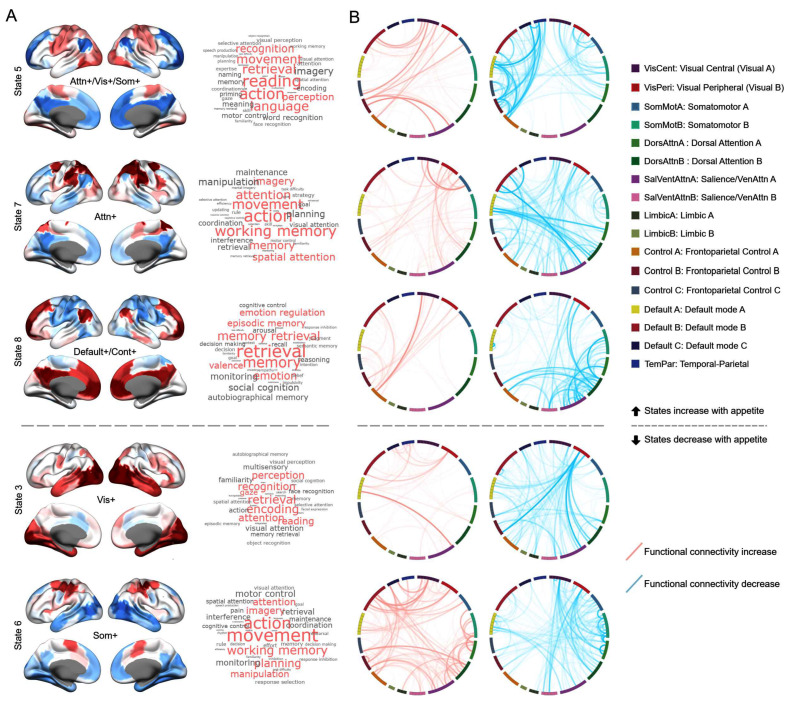
Brain states robustly associated with appetite scores. (**A**) Activation maps of the HMM inferred brain states. The word clouds on the right represent meta-analytic decoding results derived from the NeuroSynth database. The terms (FDR, *q* < 0.05) illustrate the potential functional roles of the associated brain regions as identified in prior studies. (**B**) Functional connectivity patterns of brain states. Pairwise functional connectivity is categorized according to the 17 networks defined by the Schaefer-2018 atlas. For visualization purposes, one-sample *t*-tests are conducted to compare each functional connection of a given state with the corresponding connections in other states, highlighting significantly increased or decreased connections (FDR, *q* < 0.05).

Moreover, we revealed that the brain states significantly predicting appetite scores exhibited differences in both activation and functional connectivity patterns (Figure 3). The FO value of two brain states involving dorsal attention network activation was found to positively predict appetite levels. One of these states (State 5) was characterized by the co-activation of unimodal networks and the dorsal attention network, accompanied by increased functional connectivity strength between unimodal and transmodal cortical networks. The other state (State 7) was primarily defined by the activation of the dorsal attention network and enhanced functional connectivity between this network and other brain networks. In addition, appetite scores were positively correlated with the activation state of the default mode and frontoparietal networks (State 8). In this state, the connectivity strength between the default mode network and the frontoparietal network increased, alongside enhanced connectivity between the frontoparietal network and unimodal networks. In contrast, states characterized by active unimodal networks (States 3 and 6) were negatively correlated with appetite scores.

### 3.3. A Certain Brain State Is Highly Selective to Food-Cue Processing

**Figure 4:**
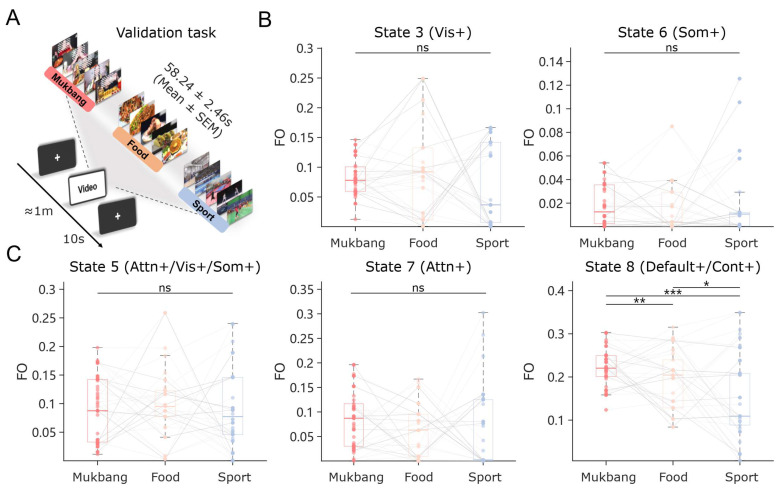
Validation of state features based on different experimental paradigms. (**A**) A task paradigm involving three types of short videos. (**B**) Repeated Measures ANOVA on states with FO negatively correlated to appetite scores in long videos. (**C**) Repeated Measures ANOVA on states with FO positively correlated to appetite scores in long videos. * *p* < 0.05, ** *p* < 0.01, *** *p* < 0.001, ns = not significant.

As high-appetite scenes may evoke more positive emotional experiences [3], an additional task was conducted to further validate that the revealed state features are specific to appetite and not predominantly influenced by other factors. To this end, the same group of participants was recruited to perform a short video-watching task. The videos included three types: Mukbang, food presentation, and sport (Figure 4A). Different experimental materials evoked varying levels of food cravings (Supplementary Figure S5; Repeated measures ANOVA: (*F* = 81.4, *p* < ·0.001)). Mukbang videos induced significantly stronger cravings than sports content (*t* = 11.4, *p* < 0.001), as well as food presentation materials (*t* = 11.3, *p* < 0.001). However, there was no statistically significant difference in food cravings between Mukbang and food presentation (*t* = −0.17, *p* = 0.99). Meanwhile, there were no significant differences in emotional arousal or valence among the short video types (arousal: *F* = 1.1, *p* = 0.303; valence: *F* = 1.3, *p* = 0.273).

To validate the effectiveness of state characteristics in an independent paradigm, we applied the previously trained HMM from the video viewing task to the current task, thereby extracting the corresponding brain state FO values. The repeated measures ANOVA revealed that the two states negatively correlated with appetite scores in prior video viewing tasks do not exhibit significant differences across the three types of short videos (Figure 4B; State 3: *F* = 1.7, *p* = 0.197; State 6: *F* = 1.6, *p* = 0.215). This result suggests that the association between these states and appetite scores may be influenced by other factors. For example, compared to scenes such as street views, high-appetite imagery may be more likely to induce higher levels of attention. In the states positively correlated with appetite scores, the FO value of State 8 (Default+/Cont+) exhibited significant differences across categories (Figure 4C; *F* = 15.6, *p* < 0.001). Specifically, the pattern of differences was as follows: Mukbang > Food (*t* = 3.12, *p* < 0.005), Mukbang > Sports (*t* = 5.03, *p* < 0.001), and Food > Sports (*t* = 2.89, *p* < 0.05). However, the other two states did not exhibit similar differences (State 5: *F* = 0.7, *p* = 0.497; State 7: *F* = 2.3, *p* = 0.106).

This finding suggests that the co-activation of the default mode network and the frontoparietal network (hereafter referred to as Default+/Cont+) is specifically associated with the processing of appetite-related information. Even when compared to sports videos with similarly high levels of emotional arousal and valence, food-related imagery consistently elicited greater state activity. Notably, Default+/Cont+ showed higher activity levels in Mukbang videos featuring human participation compared to videos with a simple display of food, despite the absence of significant differences in food craving between the two video types.

### 3.4. State Expression Associates with BMI and Food Craving

**Figure 5:**
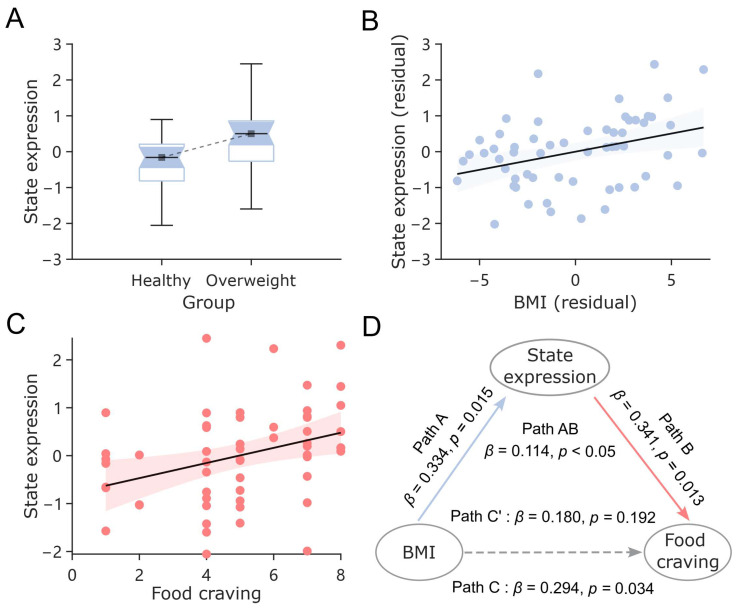
The relationships between state expression, BMI, and food craving. (**A**) Differences in state expression between the overweight/obese group and the healthy-weight group. (**B**) The linear relationship between state expression and BMI. The values on the *X*-axis and *Y*-axis represent the residuals of state expression and BMI, respectively, after controlling for age. (**C**) The linear relationship between state expression and food craving. The solid line indicates the linear regression fit, and the shaded region represents the 95% confidence interval of the regression estimate. (**D**) The mediating role of state expression in the relationship between BMI and food craving. The solid lines indicate significant pathways, while the dashed line represents a non-significant one.

Having established the robust relationship between the FO of Default+/Cont+ and appetite scores, we next aimed to investigate whether this state’s covariation with appetite is linked to BMI and food craving. Here, we defined the state expression of Default+/Cont+ associated with increased appetite by examining the correlation between FO values and appetite scores. A higher state expression reflects a stronger synchronization between the increase in FO values and rising appetite scores (Figure 5).

Between-group comparisons revealed that the Default+/Cont+ state expression was stronger in the overweight/obese group compared to the healthy-weight group (*t* = −2.16, *p* < 0.05). State expression was also found to be linearly correlated with BMI (*r* = 0.32, *p* < 0.05), and this relationship remained significant after controlling for age effects (*r* = 0.35, *p* < 0.005). Beyond BMI, the state expression of Default+/Cont+ was positively correlated with post-viewing food craving (*r* = 0.40, *p* < 0.005) and fully mediated the relationship between BMI and food craving (*r*^2^ = 38.78%, adjusted for age).

Collectively, these findings suggest that individuals with higher BMI are more likely to exhibit increased Default+/Cont+ state expression during high-appetite segments, which in turn contributes to greater food craving.

### 3.5. State Expression Differentiates Future Trajectories of BMI Change

**Figure 6:**
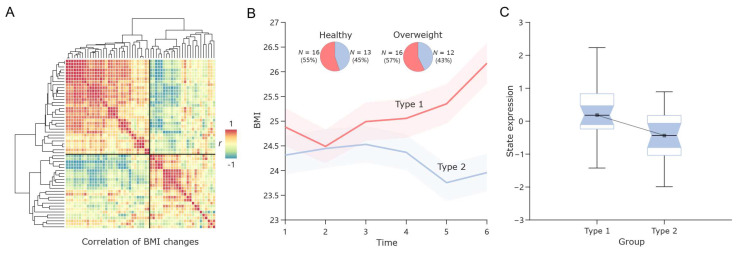
The predictive role of state expression in different BMI change trajectories. (**A**) Hierarchical clustering based on BMI change trends. The heatmap illustrates the Pearson correlations between participants’ BMI patterns across six time points. The branch lengths in the dendrogram represent the differences in BMI change trends between participants. Longer branches indicate greater differences. (**B**) The two types of BMI changes. The solid lines represent the mean BMI at each time point, while the shaded areas indicate the 95% confidence intervals (CIs). The pie chart illustrates the proportions of participants from the overweight/obese group and the healthy-weight group within each type. (**C**) Differences in state expressions between the two types of BMI changes.

Finally, we attempted to investigate whether the state expression of Default+/Cont+ could also reflect future BMI change trends. To this end, we employed hierarchical clustering to categorize participants into distinct groups based on their unique BMI change trajectories over the subsequent six months (Figure 6A). Evaluation using the Davies–Bouldin index and Calinski–Harabasz index suggested that the BMI change patterns of the participants could be broadly classified into two types (Figure 6B and Supplementary Figure S6). In one type (Type 1), BMI gradually increased over time, while in the other (Type 2), BMI remained relatively stable during the first four months but showed a slight decline in the final two months. The proportions of the two types were roughly similar between the overweight/obese group and the normal-weight group, with a slightly higher number of participants belonging to the first type compared to the second. Independent sample *t*-test revealed that participants in Type 1 (BMI increase group) exhibited significantly higher state expression of Default+/Cont+ compared to those in Type 2 (*t* = 2.28, *p* < 0.05).

The findings indicate that the state expression of Default+/Cont+ in response to food-cue videos is not only linked to current BMI and food craving but also serves as a predictor of future BMI change trends. Specifically, higher state expression is associated with a greater likelihood of BMI increase over time.

## 4. Discussion

This study, grounded in a naturalistic food-cue video paradigm, employed fMRI to dynamically investigate the brain state characteristics associated with appetitive processing in 58 female participants. Using two long food-cue videos with appetite ratings, we applied HMM and machine learning-based regression to identify brain states that reliably predict appetite fluctuations during video viewing. To validate the specificity of these states to food-cue processing, we employed an independent short-video task paradigm consisting of three conditions: mukbang, food presentation, and sport. Cross-validation between the two long-video datasets, along with external validation using an independent paradigm, revealed that activation states within the default mode and frontoparietal networks are consistently associated with appetitive processing. Specifically, the proportion of these states increases as the appetite scores of the video segments rise, and these states are more prevalent in food-related videos compared to sport videos. We further identified appetite-related state expression based on the correlation strength between state proportions and appetite scores. This measure showed significant positive correlations with participants’ BMI and post-viewing food cravings, fully mediating the relationship between the two. Moreover, appetite-related state expression predicted participants’ BMI trajectories over the following six months. Participants in the BMI growth group exhibited significantly higher expression of this state compared to those in the BMI maintenance group. These findings provide novel insights into how the brain processes food cues in naturalistic contexts and offer potential neural markers for identifying individuals at risk for weight gain.

In recent years, naturalistic paradigms have emerged as a transformative methodology in psychological and neuroscience research, offering unprecedented insights into human cognition and behavior. In contrast to conventional experimental paradigms, naturalistic approaches, particularly video-based stimuli, provide superior ecological validity, enabling researchers to capture cognitive processes as they unfold in real-world contexts [17,20]. These paradigms excel in elucidating both collective neural activity patterns [52] and individual-specific variations in cognitive processing of identical stimuli [53,54]. Despite substantial progress in various research domains, the application of naturalistic paradigms to the study of eating behaviors and obesity remains limited. Given the complexity of food-cue processing [55], traditional experimental paradigms may fail to evoke authentic appetite-related experiences, imposing constraints on uncovering the underlying neural mechanisms. This study uses ecologically valid food-cue videos as stimuli to record neural activity during viewing, marking a pioneering effort in the field. A distinctive feature of our approach lies in the utilization of continuous subjective appetite ratings, enabling more nuanced analysis of dynamic brain activity patterns compared to conventional categorical methodologies [56].

Employing food-cue videos, we revealed that active states within the default mode and frontoparietal networks are closely linked to appetitive processing. The proportion of these states shows a positive correlation with appetite scores and is significantly higher during food-related scenes compared to non-food scenes. The appetite-related brain state primarily highlighted regions within the default mode network, including the precuneus/posterior cingulate cortex (PCC), temporoparietal junction (TPJ), and ventromedial prefrontal cortex (vmPFC), as well as regions within the frontoparietal network, such as the dorsomedial prefrontal cortex (dmPFC). The neural activity in these brain regions plays a critical role in higher-order cognitive functions such as value evaluation [57], self-referential processing [58], episodic memory [59], and appetitive processing [60]. Interestingly, the cognitive functions associated with these brain regions are closely linked to memory-related processing. Their prominent involvement in food-cue processing aligns with recent perspectives suggesting that mammalian memory systems may have evolved to prioritize the retention of eating events and food-related information, playing a crucial role in food-related cognitive activities [55]. Memory plays a crucial role in food-cue processing by influencing the perception, evaluation, and decision-making processes related to food. Specifically, memory systems integrate past experiences with food (e.g., taste, texture, and reward value) to shape current responses to food cues and guide future eating behaviors [49]. Episodic memory, which encodes specific food-related experiences, contributes to the recognition of familiar foods and the anticipation of their associated rewards [61]. For instance, recalling the pleasurable experience of eating a specific food can enhance craving and appetite when encountering similar cues. Additionally, semantic memory, which stores general knowledge about food and nutrition, helps individuals evaluate food choices based on learned information about their health or caloric content [12]. Neural activity in the default and frontoparietal networks not only facilitates the retrieval of past food-related memories but also likely supports the generation of new mental imagery associated with food-cue processing [62]. Mental imagery, involving the “top-down” reactivation of sensory inputs, is believed to enhance adaptive behavior by simulating future actions based on prior experiences [51,63]. This emphasizes the crucial role of imaginative eating in shaping appetitive processing [64].

In addition, a brain state characterized primarily by dorsal attention network activation was also significantly positively correlated with appetite scores. On one hand, the network activity pattern of this state may be associated with top-down attentional orientation toward external stimuli. Orienting attention triggered by external stimuli helps individuals maintain coherent and effective cognitive processing in complex and dynamic environments. As a result, enhanced activity in the dorsal attention network has been observed across various types of salient stimuli [65]. Notably, no differences in this state were found between motion and food-related videos, supporting the notion that the brain processes salient stimuli in a domain-general manner rather than being specific to food-related cognitive processing. On the other hand, this study also observed that activation states in the sensorimotor and visual cortices decreased as appetite increased. This may be related to the competition and allocation of attentional resources. When food stimuli are presented, the brain allocates more attentional resources to multimodal food-related processing, such as integrating sensory information about the appearance, smell, taste, and caloric content of food. This may reduce the processing resources available for single modalities, such as vision, leading to decreased activity in the associated brain regions [66]. However, the suppression of these unimodal network states was also not confirmed to be food-specific and, therefore, cannot be considered a key neural marker of appetitive processing.

Importantly, the food-cue video task effectively captured individual differences related to overweight/obesity and food craving. Specifically, a stronger positive correlation between the proportion of activation states in the default and frontoparietal networks and appetite scores (i.e., the state expression of appetitive processing) was associated with higher BMI, distinguishing overweight/obese individuals from those with a healthy weight. Furthermore, the state expression of appetitive processing linked to BMI significantly influenced post-viewing food craving, with greater state expression leading to stronger cravings. These findings align with previous research showing that individuals with obesity exhibit heightened neural responses to food cues in regions associated with reward processing and executive control [67,68]. Furthermore, our findings build upon previous research by showing that these neural signatures not only distinguish weight status but also predict the intensity of subsequent food cravings. This supports the idea that altered neural responses to food cues play a role in sustaining obesity by amplifying cravings, which may, in turn, drive subsequent eating behaviors [1].

Notably, the expression of appetite-relevant states was associated not only with current BMI but also with predicted trends in BMI changes over the following six months. Compared to individuals who maintained a stable BMI, those with gradually increasing BMI exhibited greater state expression. This longitudinal relationship provides compelling evidence that neural responses to food cues may serve as potential biomarkers for identifying individuals at risk for future weight gain. This finding aligns with previous longitudinal studies showing that heightened neural responsivity to food cues predicts future weight gain [51,69] and extends this work by demonstrating that such predictive relationships are observable in more naturalistic viewing conditions. These findings have important implications for early intervention strategies, suggesting that therapeutic approaches targeting the modulation of appetite-relevant neural networks might be particularly beneficial for individuals showing elevated state expression patterns, even before significant weight gain occurs.

Several limitations of the present study warrant consideration and suggest directions for future research. First, our experimental paradigm employed a relatively homogeneous set of video stimuli with consistent stylistic features. Future studies should incorporate more diverse stimuli, such as movie clips, and integrate real-time behavioral responses (e.g., button presses) during task execution to obtain more fine-grained measures of appetitive responses. Second, while naturalistic stimuli like video clips offer ecological validity, they inherently contain complex information including emotional content, semantic knowledge, and social interactions. The current study did not explicitly control for or examine these potential confounding factors. Future investigations would benefit from more sophisticated stimulus coding schemes that enable the systematic analysis of these multiple dimensions of influence. Third, as our study was conducted exclusively with female participants, the generalizability of our findings to male populations remains to be established, and potential gender-specific modulatory effects need to be examined in future research. Although women exhibit higher sensitivity to visual food stimuli than men, meta-analytic evidence indicates that men show similar cue-evoked activation in reward-related brain regions, albeit with smaller effect sizes [24,25]. We therefore expect that similar dynamic neural features may characterize appetite in both sexes, though with attenuated responses in men. Fourth, our findings fundamentally describe correlational relationships between neural dynamics and appetite fluctuations rather than establishing causal mechanisms. Future research employing experimental manipulations is needed to determine whether these neural states directly drive appetite changes or merely reflect downstream consequences of other regulatory processes. Finally, while naturalistic paradigms show considerable promise for clinical applications [17], further research is needed to extend these findings to special populations. Specifically, investigating brain dynamics in individuals with eating disorders could provide valuable insights for developing personalized interventions and treatments in clinical settings.

## 5. Conclusions

In conclusion, by employing dynamic neuroimaging techniques combined with ecologically valid food-cue videos, we identified distinct neural states primarily involving the default mode and frontoparietal networks that sensitively reflect moment-to-moment fluctuations in appetite. These neural patterns effectively differentiated individuals according to their current weight status and showed promising associations with longitudinal changes in BMI, suggesting their potential utility as early biomarkers for obesity risk. However, the predictive value of these neural markers requires further validation in larger and more diverse samples before clinical applications can be considered. Beyond their biomarker potential, our findings clarify the dynamic neural mechanisms underlying consumer responses to naturalistic digital food cues and provide valuable insights into how daily media exposure influences eating behaviors and weight management.

## Data Availability

The original contributions presented in this study are included in the article/Supplementary Materials; further inquiries can be directed to the corresponding author.

## Supplementary Materials

The following supporting information can be downloaded at: https://www.mdpi.com/article/10.3390/nu17152449/s1, Figure S1: Split-half analyses demonstrated that the rated appetite scores for each video were consistent across participants; Figure S2: Evaluation of the optimal number (K) of brain states; Figure S3: Brain states uncovered by Hidden Markov Model (HMM); Figure S4: Under varying parameter conditions, we validated the data for each individual in both video datasets using the five selected brain state features; Figure S5: Comparison of subjective ratings across three dimensions for different types of short videos; Figure S6: Evaluation of the optimal number of BMI change types.
