# Supplementary material for: Seeing Is Craving: Neural Dynamics of Appetitive Processing During Food-Cue Video Watching and Its Impact on Obesity
Source: Nutrients. 2025 Jul 27;17(15):2449. doi: 10.3390/nu17152449 (PMC12348618; doi:10.3390/nu17152449)
Supplement: Supplementary file 1 [file nutrients-17-02449-s001.zip › nutrients-3759070-supplementary.pdf]

Supplementary Materials

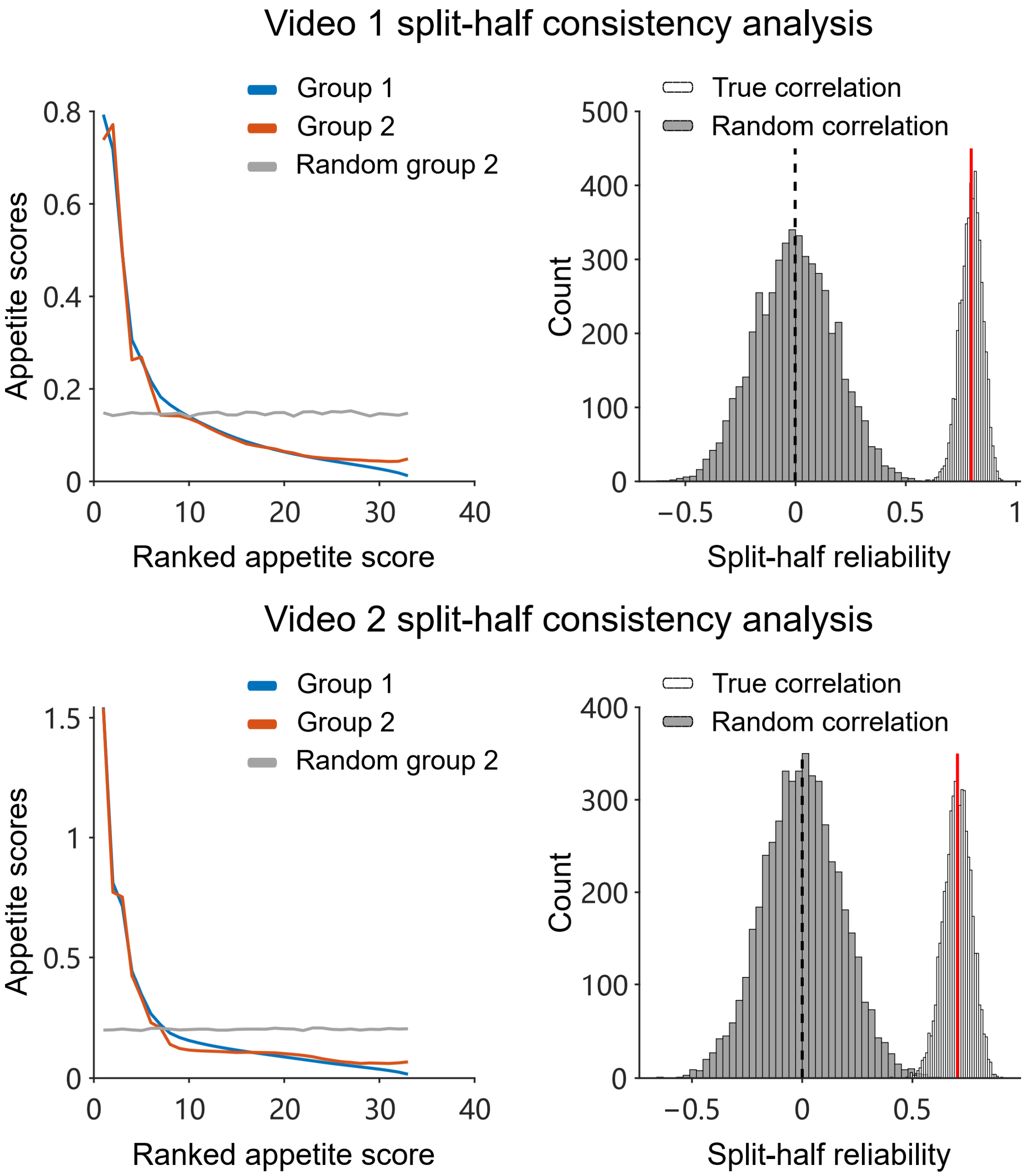

Supplementary Figure S1. Split-half analyses demonstrated that the rated appetite scores for each video were consistent across participants. Left panel: The blue line represents the averaged appetite scores ranked for a random split-half group (Group 1), while the orange line represents the averaged appetite scores of the remaining half (Group 2), ranked based on the data from Group 1. Both lines reflect averages across 5,000 iterations. The grey line represents an estimation of chance level, obtained by shuffling the rank orders of Group 2 (surrogate data). Right panel: The mean split-half correlation coefficients across 5,000 iterations were significantly higher than the surrogate (null) distributions for both video datasets. The red line indicates the mean of the split-half correlation coefficients across 5,000 iterations, while the black dashed line represents the zero correlation point.

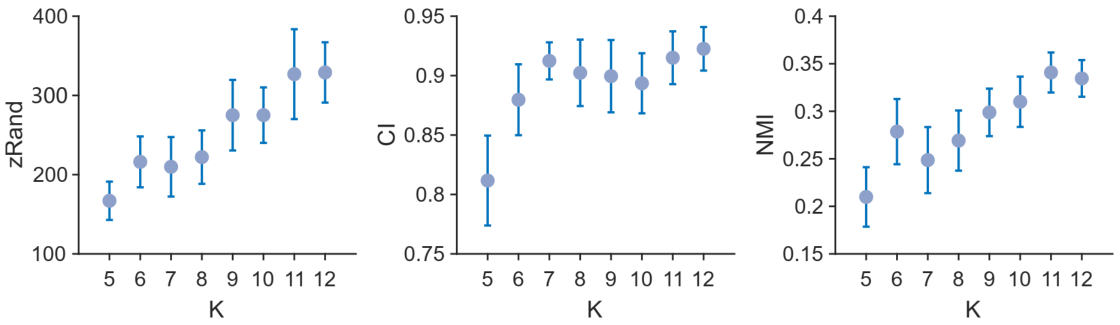

Supplementary Figure S2. Evaluation of the optimal number (K) of brain states. The optimal number of states in the HMM was evaluated based on the model's generalization performance across split-half data. The model's generalization performance was evaluated using three metrics. zRand index: The zRand index is a normalized version of the Rand index, specifically designed to measure the similarity between two classification results. A higher zRand value indicates greater similarity between the two classification schemes. By normalizing random pairings, zRand eliminates biases caused by differences in class sizes, providing a more accurate measure of consistency between classification results. Concordance index (CI): The CI metric is used to evaluate the concordance of activation patterns under the same brain state across two independent datasets. A CI value closer to 1 indicates higher consistency in activation patterns between datasets within the same brain state. Normalized mutual information (NMI): Based on information theory, NMI evaluates the amount of shared information between two classification schemes. A higher NMI value reflects stronger correlation between the two classifications. Unlike zRand, NMI quantifies the overlap of classification results at the information level by calculating mutual information. NMI values range from 0 to 1, where a value of 1 indicates perfect agreement between the two classification schemes.

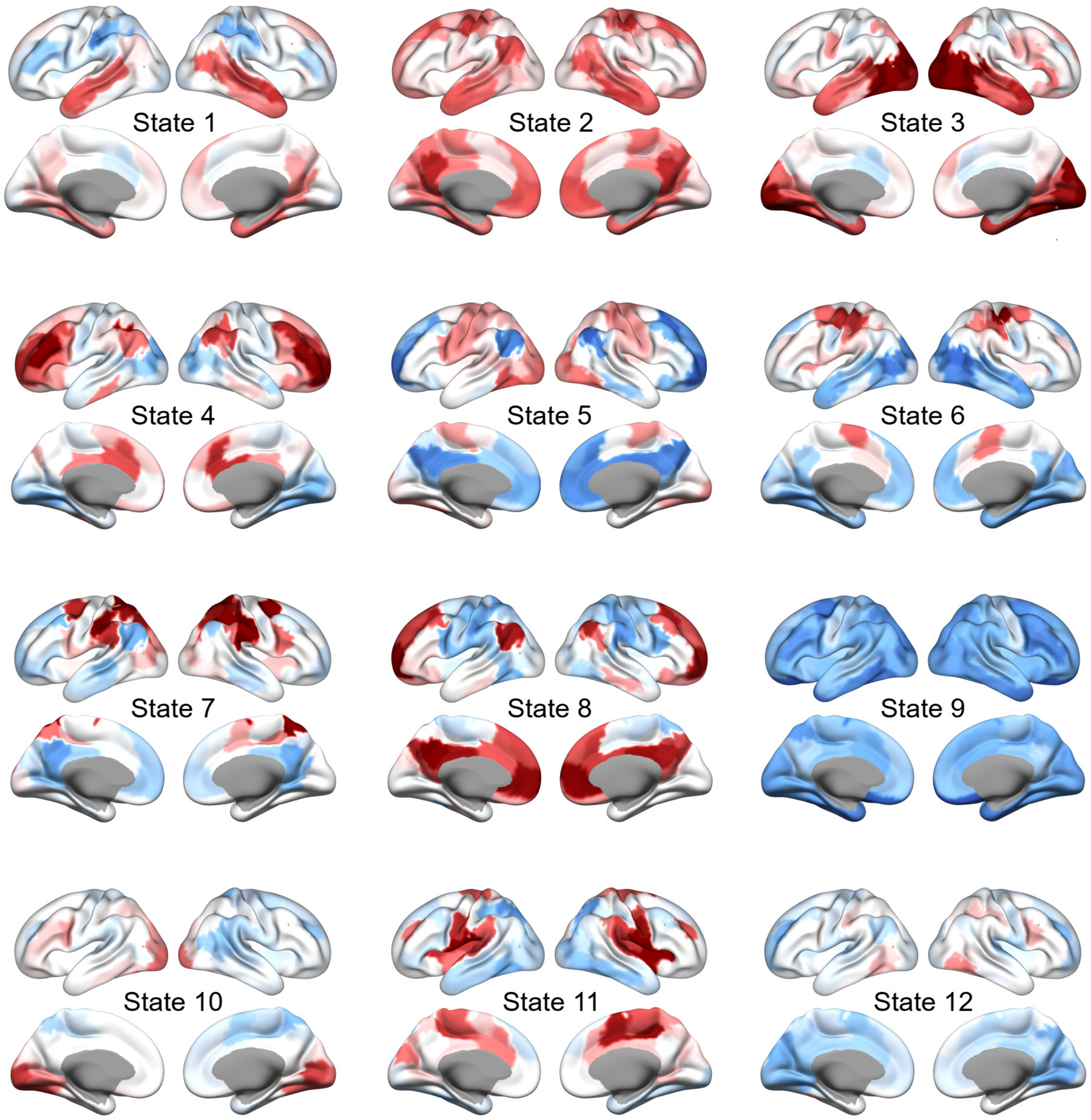

Supplementary Figure S3. Brain states uncovered by Hidden Markov Model (HMM). Using HMM inference on the concatenated fMRI time series of participants watching two long videos, we identified 12 distinct brain states that represent structured, recurrent patterns of functional activation. State 1 was characterized by widespread activation in the temporal lobe, while State 2 exhibited broad positive activation across multiple brain regions. State 3 was dominated by activation in the visual cortex, with additional involvement of the sensorimotor areas and temporal lobe, and State 4 was marked by activation of the frontoparietal control network. State 5 involved joint activation of the sensorimotor, visual, and dorsal attention networks, whereas State 6 was characterized by prominent activation in the sensorimotor areas near the central sulcus. State 7 reflected combined activation of the dorsal and ventral attention networks, and State 8 primarily involved the default mode network, with extensions into parts of the frontoparietal control network. In contrast, State 9 displayed widespread deactivation across large-scale brain regions, while State 10 was defined by activation in the primary visual cortex. State 11 featured joint activation of the ventral attention and sensorimotor networks, and State 12 was primarily characterized by activation in the dorsal attention network and occipitotemporal cortex. These 12 states collectively captured the dynamic and functionally organized nature of brain activity during video watching.

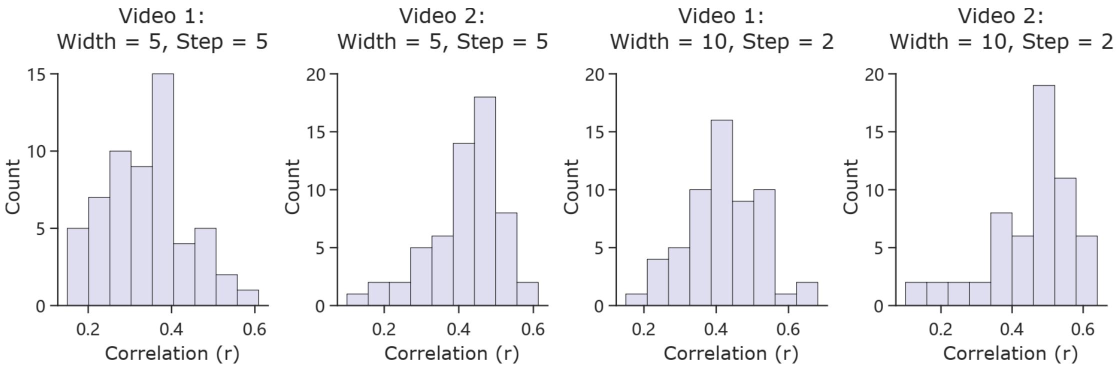

Supplementary Figure S4. Under varying parameter conditions, we validated the data for each individual in both video datasets using the five selected brain state features. Video 1 (width = 5; step = 5): $r = 0.34 \pm 0.01$ (mean ± standard error); Video 2 (width = 5; step = 5): $r = 0.41 \pm 0.01$. Video 1 (width = 10; step = 2): $r = 0.49 \pm 0.02$ (mean ± standard error); Video 2 (width = 10; step = 2): $r = 0.38 \pm 0.01$.

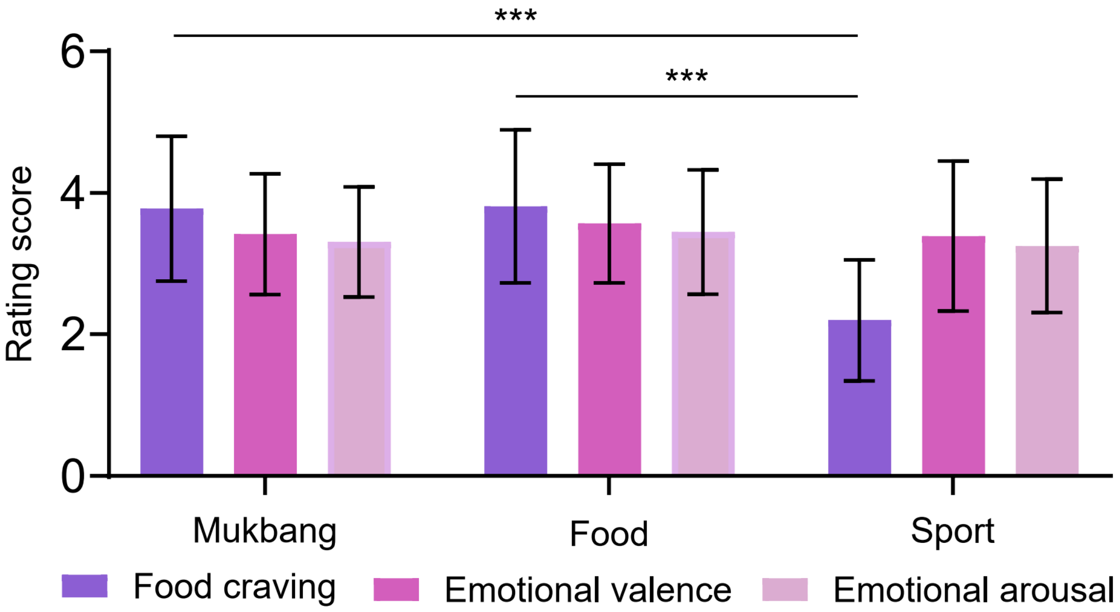

Supplementary Figure S5. Comparison of subjective ratings across three dimensions for different types of short videos. *** $p < 0.001$.

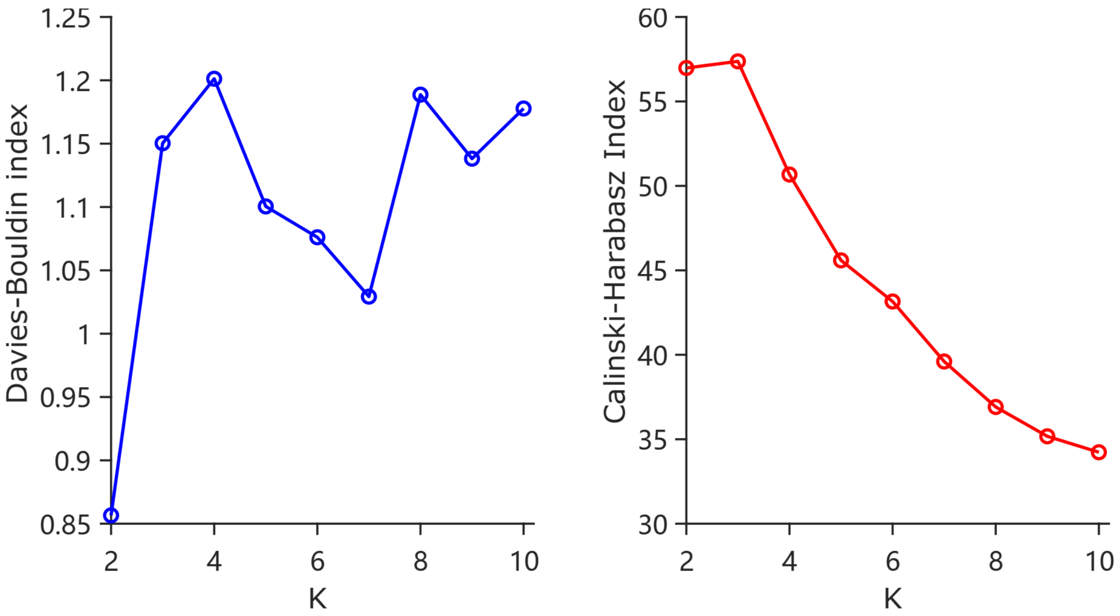

Supplementary Figure S6. Evaluation of the optimal number of BMI change types. The decision to select two clusters was supported by multiple cluster validity indices, specifically the Davies-Bouldin index and the Calinski-Harabasz index, which are widely used to evaluate the quality of clustering solutions. The Davies-Bouldin index, which measures the average similarity ratio of each cluster with its most similar cluster (where lower values indicate better clustering), exhibited a local minimum at two clusters, suggesting improved separation and compactness compared to other configurations. Meanwhile, the Calinski-Harabasz index, which assesses the ratio of between-cluster dispersion to within-cluster dispersion (where higher values indicate better-defined clusters), reached its peak value at the similar level, further confirming that the two-cluster solution provides a good balance between cluster separation and internal cohesion. Together, these indices strongly validated the selection of two clusters as the most appropriate configuration for categorizing the types of BMI changes.
